# Boost your brain, while having a break! The effects of long-term cognitively engaging physical activity breaks on children’s executive functions and academic achievement

**DOI:** 10.1371/journal.pone.0212482

**Published:** 2019-03-06

**Authors:** Fabienne Egger, Valentin Benzing, Achim Conzelmann, Mirko Schmidt

**Affiliations:** Institute of Sport Science, University of Bern, Bern, Switzerland; UNSW Sydney, AUSTRALIA

## Abstract

Classroom-based physical activity (PA) is gaining attention in terms of its potential to enhance children’s cognitive functions, but it remains unclear as to which specific modality of PA affects cognitive functions most. The aim of the study was to examine the effects of qualitatively different PA breaks on children’s cognitive outcomes. Children (*N* = 142) aged between 7 and 9 years were allocated to a 20-week classroom-based PA program, with either high physical exertion and high cognitive engagement (*combo group*), high physical exertion and low cognitive engagement (*aerobic group*), or low physical exertion and high cognitive engagement (*cognition group*). Executive functions (updating, inhibition, shifting) and academic achievement (mathematics, spelling, reading) were measured pre- and post-intervention. Results showed that the combo group profited the most displaying enhanced shifting and mathematic performance. The cognition group profited only in terms of enhanced mathematic performance, whereas the aerobic group remained unaffected. These results suggest that the inclusion of cognitively engaging PA breaks seem to be a promising way to enhance school children’s cognitive functions.

## Introduction

There is a growing body of research supporting a positive relationship between physical activity (PA), cognitive functions and academic achievement [1–4]. Cognitive functions, particularly executive functions (EFs), are acknowledged as a predictor for academic achievement [5]. The term “EFs” refers to a set of top-down mental processes that allows for controlled and goal-directed behaviour [6]. EFs can be subdivided into three core dimensions: The first dimension is *updating*, the ability to keep relevant information in working memory. The second dimension, *inhibition*, refers to the avoidance of dominant, automatic or prepotent responses. The third dimension, *shifting*, is based on updating and inhibition, and represents the ability to change among multiple tasks, operations, rules or perspectives. From a developmental perspective, inhibition is the first EF to be fully developed in children, whereas shifting is the last [5]. In general, high levels in EFs predict school readiness in young children [7] and explain a substantial amount of variance in elementary school children’s academic achievement [8]. Although not deriving from interventional studies, longitudinal data supports the mediating role of EFs between PA and academic achievement [9, 10].

Research has shown that long-term PA programs have a positive effect on the EFs and on academic achievement [3, 4]. Several physiological mechanisms have been proposed to explain these positive effects, including exercise-induced neurogenesis, angiogenesis, enhanced nervous system metabolism and increased catecholamine neurotransmission [11, 12]. EFs in turn seem to play a mediating role in the relationship between PA and academic achievement [10, 13, 14], indicating that increased PA promotes motor abilities and better EF performance, which consequently impacts academic achievement. Thus, specifically designing physical activities to infuse them into the school setting might be a cost-efficient way to enhance both children’s EFs and at the very end their academic achievement.

Recently, intervention studies are revealing that not all forms of PA benefit cognition equally. Besides quantitative aspects (such as exercise duration and intensity), qualitative aspects (such as exercise type) have also been shown to affect children’s EFs [15]. To date, the cognitive engagement (CE) inherent to many forms of PA is one of the qualitative aspects most widely discussed [16]. Increased cognitive demand is thought to induce CE, which is defined as the degree to which cognitive effort is needed to master difficult skills [17]. The “cognitive stimulation hypothesis” [18] provides a possible explanation for the cognitive improvement achieved through the cognitive demands inherent to PA exercises. The assumption is that cognitively demanding exercises activate similar brain regions used to control higher-order cognitive processes [16, 19]. The activation of these specific regions, through the participation in cognitively demanding exercises, leads to cognitive benefits in the circumscribed domains of executive functioning [15, 20].

Considering the existing literature, the current results seem to support this theoretical assumption. Vazou, Pesce, Lakes and Smiley-Oyen [21] found a medium effect of cognitively engaging PA on cognitive outcomes in their meta-analyses. Interventions comparing cognitively challenging vs. cognitively non-challenging PA, found the enhancement to be significantly more pronounced in response to cognitively engaging activities [22, 23]. It is evident that the interest in cognitive engagement during PA is gaining attention, however, research into PA modalities and their impact on cognitive outcomes is still in its infancy. Furthermore, several limitations make it difficult to draw comparisons between existing studies [21]. The high heterogeneity within the same categories of PA programs aggravated a clear understanding of effectiveness. Firstly, it is possible that programs defined as one specific modality of PA include substantially different contents and PA characteristics. Secondly, within a specific PA program several PA characteristics can be responsible for the cognitive stimulation induced. Thirdly, the reliability by which a PA program is implemented in practice can influence its efficacy and effectiveness. Consequently, there is a need to systematically vary the amount of CE inherent in PA exercises. For example, one study which varied the amount of CE systematically found positive effects, only through the implementation of cognitively enriched PA. Cognitively enriched PA games, such as basketball and floorball, elicited cognitive improvements in 10 to 12-year-old children, whereas both the mostly aerobic intervention (running exercises) and a traditional physical education curriculum, including only few cognitive demands, showed no beneficial effects on children’s cognitive outcomes [23]. Thus, it seems plausible that cognitively engaging long-term PA interventions provide an opportunity to modulate children’s cognitive functions effectively.

Classroom-based PA seems to be a promising tool to enhance not only daily PA, but also EFs and academic achievement [24]. Recently, studies have applied chronic classroom-based interventions and found encouraging results. These benefits range from enhanced classroom behavior [25–27] to improved academic achievement [28–30]. In general, two different types of (long-term) classroom-based PA can be distinguished: a) integrated PA, which incorporates PA *during* academic lessons (e.g. hopping the result of an arithmetic problem) and b) PA breaks, which consist of short bouts of PA *between* lessons (e.g. performing coordinative exercises) [31]. In a large study, including 1322 participants, improved classroom behavior was observed after 8 months of daily implemented PA breaks [25]. In another large long-term intervention, no improvements in academic achievement through daily PA breaks over 3 years were found, but academic achievement outcomes did not diminish compared to the control group [32]. This suggests that additional PA time in classroom, at least, has no negative influence on academic achievement.

A closer examination of studies finding positive effects on children’s cognitive outcomes through classroom-based PA uncovers differences concerning the specific PA modality implemented. Most classroom-based interventions featured basic aerobic movements (jogging, hopping, skipping) [27, 33, 34], whereas in others specific coordinative movements, like rope skipping or dancing had to be performed [35]. However, if experimental conditions were mostly compared to an passive control condition, e.g. sedentary academic or regular lessons, it is not surprising that children’s cognitive functions benefit from all varieties of PA interventions when they were compared to either no treatment or purely academic content. With the aim of systematically varying the amount of CE inherent in PA exercises, more than two groups including an active control group seem to have several advantages: improvements in academic related outcomes can be traced back as a result of the PA breaks intervention and shed light on a specific underlying mechanism. Further, active control groups deal better with confounds such as motivation, treatment credibility and internal validity [36].

Most previous studies either targeted children’s EFs [3] or academic achievement [30, 33, 35, 37, 38] as a separate outcome. Bearing in mind the aforementioned connection between PA and academic achievement with EFs playing a mediating role, only few studies [10, 39] included the core EFs and academic achievement as linked outcomes in the same design. Egger, Conzelmann and Schmidt [40] disentangled the separate and- /or combined short-term effect of physical exertion and CE induced by a single bout of classroom-based PA on children’s EFs. Consequently, there is another need to systematically differing between the long-term effects of cognitively engaging PA breaks, comparing them to active controls with less CE or less physical exertion.

The aim of the present study was to investigate the effects of three qualitatively different long-term PA break interventions, each with diverging quantities of cognitive engagement and physical exertion on both primary school children’s three core EFs and their academic achievement. It was hypothesized that PA breaks that combine physical effort with high cognitive demands are more effective than PA breaks with either low cognitive demands or low physical effort. Therefore, the effectiveness of three PA break interventions were compared: (1) The *combo group* with high amounts of both cognitive engagement and physical exertion, (2) the *aerobic group* with low cognitive engagement and high physical exertion, and (3) the *cognition group* with high cognitive engagement and low physical exertion.

## Materials and methods

### Design

The parents of the children provided written informed consent to participate in this study. Discontinuation was possible at any point during the study, and all data were treated with strict confidentially. The Institutional Review Board of the Faculty of Human Sciences at the University of Bern has approved the study protocol prior the enrolment of the first participant.

Three 20-week interventions with different amounts of cognitive engagement and physical exertion were compared with respect to their effects on children’s EFs and academic achievement. The intervention duration of 20 weeks was chosen in order to follow recommendations of a recent meta-analysis indicating that the longer the intervention, the greater the effects on cognitive performance [41]. Furthermore, interventions were limited to 20 weeks because they had to fit into a school phase, which was not interrupted by holiday, in order to control confounding effects of the holiday activities.

Altogether, twelve classes were randomly assigned to one of the three experimental conditions: (1) The *combo group* with high levels of both cognitive engagement and physical exertion, (2) the *aerobic group* with low cognitive engagement and high physical exertion, and (3) the *cognition group* with high cognitive engagement and low physical exertion. The teachers were informed about the basic aims of the study but were blinded with respect to the specific hypotheses. Nevertheless, in each condition, the teachers were told that a positive effect on cognitive performance was expected. As usual in classical comparison pretest-to-posttest designs, each intervention was both preceded and followed by a measurement point for data collection (of the dependent variables: EFs and academic achievement). Prior to the intervention, information about age, gender, PA level, and socioeconomic status was collected using questionnaires. Height and weight (for calculating the body mass index, BMI), aerobic fitness and gross motor coordination were determined using standardized measures and tests. For the manipulation check, children’s step counts were measured using accelerometers throughout one specific hour (10:30 a.m.– 11:30 a.m.) over five consecutive school days. It was ensured that the PA break was implemented during this hour.

### Participants

Based on a recent meta-analysis in children targeting EFs [41], a small to moderate effect (*f* = .12) of classroom based physical activity breaks on the core EF was expected. A priori power analysis using G*Power [42] with power (1- β) set at 0.80 and α = .05 showed that a sample size of *N* = 138 was requested to reach statistical significance at the .05 level. To recruit participants for the current study, teachers and headmasters were directly contacted. Altogether, 142 participants ranging from 7 to 9 years (*M* = 7.91 years, *SD* = 0.40; 54.9% girls) from the region of Bern, Switzerland, were included in this study. The exclusion criteria required for participants to have no formal diagnosis of ADHD, dyslexia, dyscalculia and colorblindness. Classes were randomly assigned to conditions using randomizer.org. There was some data loss due to sickness, technical problems with the tablets, or non-participation in the fitness tests because of injury. The percentage of pupils with incomplete values was 3.9% at pre-test and 5.9% at post-test. Since the missing completely at random (MCAR) test according to Little [43] was not significant (χ^2^ (20) = 13.93, *p* = .834), the missing values were estimated with the help of the expectation–maximization (EM) algorithm. Hence, a complete set of data was used for the statistical analyses.

There were no significant differences between the three experimental conditions with respect to age (*F*(2, 139) = 1.76, *p* = .176, η_p_^2^ = .025), gender distribution (χ^2^(2) = 0.24, *p* = .887, Cramer’s *V* = .041), PA level (*F*(2, 139) = 2.31, *p* = .103, η_p_^2^ = .032), socioeconomic status (*F*(2, 139) = 0.90, *p* = .408, η_p_^2^ = .013), BMI (*F*(2, 139) = 0.23, *p* = .795, η_p_^2^ = .003), aerobic fitness (*F*(2, 139) = 1.90, *p* = .153, η_p_^2^ = .027) and gross motor coordination (*F*(2, 139) = 0.51, *p* = .599, η_p_^2^ = .007).

### General procedure

The interventions were carried out in the classroom by the regular class teachers, which were blinded with regard to the specific hypotheses. Two times 10 minutes sessions per day over a period of 20 weeks should be carried out, whereby the entire intervention was intended to cover 200 PA breaks. In each group, prior to the study, teachers completed a half-day training program instructing them in the basic principles, aims and purposes of the intervention program, demonstrating the specific contents with the special teaching materials. To test implementation compliance [44] teachers had to report the number of PA breaks effectively carried out, including the implementation accuracy. The teachers reported that they had implemented *M* = 145.4 of the prescribed 200 PA breaks (range between 107–172). The realized number of PA breaks during the 20 weeks did not differ between the three conditions (*M*_*cognition*_ = 151.8, *SD* = 29.7 vs. *M*_*aerobic*_ = 125.0, *SD* = 21.6 vs. *M*_*combo*_ = 157.3, *SD* = 14.6; *F*(2,7) = 1.61, *p* = .267, η_p_^2^ = .315). Concerning the implementation accuracy, teachers reported that they could implement the PA breaks at a level of *M* = 3.4 using a 4-point Likert scale (ranging from 1 = “not at all” to 4 = “exactly as the description”). The three conditions did not differ in terms of implementation accuracy (*M*_*cognition*_ = 3.6, *SD* = 0.09 vs. *M*_*aerobic*_ = 2.9, *SD* = 0.60 vs. *M*_*combo*_ = 3.5, *SD* = 0.40; *F*(2,7) = 2.36, *p* = .165, η_p_^2^ = .403).

Prior to the study, background variables were measured at the respective schools. In addition, each child completed the same EF testing twice: before (pre-test) and after (post-test) the intervention, each completed in one session. The cognitive testing took place in a quiet room in groups of four children. Two investigators, who were blinded with respect to the conditions, performed the testing. They gave general instructions whilst the children were encouraged to work quietly, but encouraged to ask questions about the test whenever something was unclear. The cognitive tasks were completed on tablets, and children received the instructions over headphones with supportive images on the screen. To complete the tasks at their own pace and without distraction, the children were seated far apart from each other. Academic achievement (mathematics, spelling, reading) was also assessed before and after the intervention, using three standardized tests. However, the tests for academic achievement were not completed on the same day as the EF testing, to reduce the impact of increased cognitive load as a confounding variable. The academic achievement testing took place as a group testing in the classroom. Two separate blinded investigators with teaching experience tested the children during one regular school lesson. The overall testing was around 45 minutes and started in every class at 10.15 a.m. After the general instructions were advocated to the class, the mathematic test started (10.15 a.m.– 10.40 a.m.). After a short break of 5 minutes, the children were tested in their spelling performance (10.45 a.m.– 10.55 a.m.), and finally in their reading performance (10.55 a.m.– 11.05 a.m.).

### Experimental conditions

*Combo group* (high cognitive engagement, high physical exertion; *n* = 47): This intervention consisted of specifically designed PA breaks tailored to challenge EFs (more information regarding the intervention can be obtained from the first author). For example, children were standing in a circle and playing the game “Horserace”. They had to run on the spot until the teacher said a key word. Whenever one of the keywords (starting with three different key words) was mentioned, the children had to react as quickly as possible with a predefined movement. For example, when they heard the word “hurdle” they had to jump up and then keep on running. The game was played for around 3 minutes, following incremental levels of difficulty (e.g. an additional keyword was defined, or the predefined movement was changed). Such rule changes intended to keep the game cognitively demanding, where the children were required to adapt their movements to the key words. Thus, the repetition with additional keywords and altered corresponding movements was crucial in this exercise. The children had to update the new information, inhibit the movements from the previous run which were no longer correct, and shift between the different words and their corresponding new movements.

*Aerobic group* (low cognitive engagement, high physical exertion; *n* = 49): This condition was designed to promote children’s aerobic fitness. Although it is not possible to exclude cognitive engagement entirely from long-term PA interventions, the attempt was made to choose exercises that had as little cognitive demand as possible. For example, the same game as mentioned in the combo group (“horserace”) was also played in the aerobic group but without the cognitive demands. The children were standing in the circle and had to run on the spot. The teacher acted out a movement (e.g. jumping) which the children had to imitate. Therefore, the children were only required to imitate the movements without remembering the correct movements relevant to the keywords. The same physical components were repeated to guarantee that PA in this group did not differ from the PA breaks in the combo intervention, regarding the physical intensity and/or the amount of social interaction.

*Cognitive group* (high cognitive engagement, low physical exertion; *n* = 46):

This intervention consisted of specifically designed breaks tailored to stimulate EFs by targeting fine motor skills. Instead of standing in a circle, the children sat in a circle and played the “horserace” game without any physical exertion. Using the same three keywords, the children were instructed to react as quickly as possible with their arms and fingers whenever they heard a keyword. For example, when they heard “hurdle”, they had to imitate a jump from a horse with their arms. After about 3 minutes, the level of difficulty was increased with either an additional keyword or by changing the matching movement, equivalent to the rules in the combo condition.

### Manipulation check variables

To test the experimental manipulation, objective as well as subjective measures were used. To assess the *physical exertion* in the three experimental conditions, children’s step counts were objectively measured using ActiGraph GT3X (ActiGraph LLC, Pensacola, FL, USA). The average number of children’s step counts and the percentage of time spent in moderate-to-vigorous intensity was calculated using the PA of one specific hour (10:30 a.m.– 11:30 a.m.) over five consecutive school days. It was ensured that the PA break was implemented during this hour in the three experimental condition.

*Perceived cognitive engagement* was measured with an adapted version of the Self-Assessment Manikin [45]. The average of 10 rated PA breaks (two PA breaks a day during five school days) was calculated. The Self-Assessment-Manikin is a widely used non-verbal pictorial assessment technique to measure a person’s affective reaction to a variety of stimuli. It consists of one item for each construct. Acceptable psychometric properties have been demonstrated by Bradley and Lang [45]. As in the original Self-Assessment Manikin, for example when judging arousal, the children had to rate their perceived cognitive engagement ranging from 1 (“not cognitively engaging at all”) to 9 (“very, very cognitively engaging”). The question they had to answer was: “how cognitively engaging was the previous activity for your brain?”. Even though the instrument has not been validated, it has been shown to be feasible in children [40] and adolescents [46].

To test whether the three conditions were comparable with respect to induced pleasure, children’s pleasure was measured using the original Self-Assessment-Manikin [45]. The mean of 10 rated PA breaks (five running school days) was calculated. Acceptable reliability and validity has been shown, with 7 to 11 year old children being able to make dimensional ratings of pleasure and arousal in ways similar to adults [47].

### Cognitive assessment

The core EFs were measured by two customized tablet-based tasks using E-Prime Software (Psychology Software Tools, Pittsburgh, PA). Each task took about 12 minutes to complete and the order of the two tasks was counterbalanced between participants.

*Updating* was assessed with the Backwards Colour Recall task [7, 48]. The task is embedded in a cover story about a dwarf who loses sequences of coloured discs, starting with a two-disc sequence. The discs were presented for 1 s, separated by interstimulus-intervals of 500 ms. The children were asked to recall the sequences in the reverse order (by pressing the correct colour disc on the tablet). The two practice blocks (two- and three-disc sequence) included 3 trials, with a feedback loop whenever 66% of the trials were incorrect. Sequence length was increased by one disc when 50% of the six trials were correct, otherwise the task was interrupted. The total score of trials recalled correctly was used as the dependent measure. Acceptable retest reliability of this measure has been demonstrated in young (4- to 5-year old) children [48].

*Inhibition* was assessed with a child-adapted version of the Eriksen flanker task [49]. The fish flanker task is considered as the child version of the Attention Network Test [50], and has widely been applied in developmental research [51–53] including exercise and cognition studies [10, 23, 54, 55]. The task consisted of two different blocks including five practice trials per block, and a feedback loop whenever the performance was below 60%. The “pure” block consisted of 16 congruent trials, and the “standard” block consisted of both 16 congruent and 16 incongruent trials, presented randomly. Inter-stimuli-intervals varied randomly from 800 to 1400 ms [51, 54]. As a dependent measure for inhibition, the conflict score between incongruent (highest rate of distraction in the standard block) and congruent trials (lowest rate of distraction in the pure block) was calculated [53, 56].

*Shifting* was assessed with an additional “mixed” block within the flanker task. A new rule cued by different coloured trials was introduced. Children had to adapt their response relating to the colour. Whenever the colour of the trials changed, a switch between the two rules was required. As before, a total of 16 congruent and 16 incongruent trials were randomly presented. Inter-stimuli-intervals varied randomly from 800 to 1400 ms [51, 54]. As a dependent variable for shifting, the global switch costs was calculated [57], determined as the difference between the mixed block and the standard block. Hence, the inhibition components within the “mixed” block were controlled (trials in the mixed block not only required shifting between different tasks, but also involve inhibitory demands).

### Academic achievement assessment

Children’s academic achievement (mathematics, spelling, reading) was assessed using three standardized academic achievement tests for second graders.

*Mathematics* performance was measured using the two subscales “arithmetic operations” and “visual-spatial functions” containing six subtests from the Heidelberger Rechentest (HRT 1–4) [58]. For each subtest a time limit between 30 s and three minutes is given. The *t*-score was calculated, which reflects the deviation of the age-related mean score. Evidence for the reliability and validity of the Heidelberger Rechentest has been provided by Haffner et al. [58].

*Spelling* was measured using the Hamburger Schreib-Probe (HSP 1–10) [59], where children are required to spell eight words and a sentence that were read aloud to them, without any time pressure. As a measure of writing, the number of correctly spelled graphemes was calculated. The *t*-scores were calculated, which reflects the deviation of the age-related mean score. Test-retest reliability for graphemes (*r*_tt_ = .97) has been shown by May [59].

*Reading* was measured using the Salzburger Lesescreening [60]. Children needed to read as many sentences as possible within 3 minutes and check if the statement is correct or not. The reading quotient, reflecting the *z*-standardized mean, was calculated. The test has been demonstrated to have acceptable reliability and validity [60].

### Background variables

The Physical Activity Questionnaire for Children (PAQ-C) [61] was used to *measure the general PA level*. The PAQ-C is a 7-day self-administered recall measure that provides a PA summary score derived from nine items. The response format varies by item, but each is scored on a 5-point scale, a sample item being: “In the last 7 days, how many times did you do sports or PA after school?”. Response options range from: “None” (1 point) to “5 times last week” (5 points). Evidence for the reliability and validity of the questionnaire in 8- to 16-year-olds has been provided by Crocker et al. [61]. The Family Affluence Scale II (FAS II) [62] was used to assess the *socioeconomic status*. The scale consists of 4 questions asking children things they are likely to know about their family (e.g. number of family-owned cars, computers, number of family holidays in the past year, and having an own bedroom at home). A sample item is: “Does your family own a car, van or truck?” Response options are: no (0 points); yes, one (1 point); yes, two or more (2 points). The response format varies by item. The prosperity index (ranging from 0 to 9) was calculated from the sum of the three items. Evidence for the reliability and validity has been provided by [63]. The BMI was calculated as the body weight (in kg) divided by the square of the height (in m).

*Aerobic fitness* was assessed using the Multistage 20 metre Shuttle Run test [64]. Participants have to run back and forth on a 20 m course as instructed by a sound signal emitted from a pre-recorded tape, ensuring they touch the 20 m line with their foot. The frequency of the sound signal increases by 0.5 km/h every minute, indicating the next stage (level), starting with a speed of 8.5 km/h. The test ends when participants fail to reach the line before the signal. Evidence for the reliability and validity of the 20 metre Shuttle Run test has been provided by Liu, Plowman, and Looney and McVeigh, Payne, and Scott [65, 66].

*Gross motor coordination*. Children’s gross motor coordination was measured using the “Körperkoordinationstest für Kinder” [67]. The children performed the four subtests: a) walking backwards b) moving sideways c) hopping for height and d) jumping sideways. Points were given for each test item to make up the overall motor quotient (MQ) under consideration of gender and age factor. The calculated internal consistency of the four tests was acceptable (α = .79). A test-retest reliability of *r* = .97 of the KTK was reported by Kiphard and Schilling [67].

### Statistical analyses

All statistical analyses were conducted using SPSS 24.0 (SPSS Inc., Chicago, IL, USA). In the *outlier analysis*, trials with a reaction time under 150 ms were excluded. In a next step, trials with reaction times deviating by more than 3 *SD* from the child’s mean were also excluded. Only correct trials were included in the calculation of reaction times. Subsequently, blocks with an accuracy of less or equal to 50% were excluded assuming that those children seemed to have either not understood the task, or to have completed it incorrectly due to a lack of motivation. In *preliminary analyses*, separate ANOVAs were completed to test: (a) the potential between-group differences in background variables (age, general PA level, socioeconomic status, BMI, aerobic fitness, gross motor coordination), (b) the between group differences in pre-test values of the dependent variables (updating, inhibition, shifting, mathematics, spelling, reading) and finally (c) the differences in manipulation check variables (mean step counts during one hour, perceived physical exertion, perceived cognitive engagement, pleasure during PA breaks) (see Table 1). Partial eta square (η_p_^2^) was reported as an estimate of effect size. When the overall ANOVA proved significance, Bonferroni-corrected post-hoc comparisons were used to determine the specific differences between the three groups. In the *main analyses*, ANCOVAs were conducted using the pre-test values of each dependent variable as covariate. The level of significance was set at *p* < .05 for all analyses. Table 2 shows means and standard deviations for accuracy and reaction times in the three core EFs at pre- and post-test for the three groups. Table 3 shows means and standard deviations for academic achievement (mathematics, spelling, reading) at pre- and post-test for the three groups.

**Table 1:** Means (and standard deviations) for the background, the manipulation check and the dependent variables in academic achievement in the three experimental conditions.

	Combo group	Aerobic group	Cognition group
**Sample characteristics**			
Age (years)	7.94 (0.40)	7.96 (0.36)	7.82 (0.41)
Gender distribution (male/female)	21/26	21/28	22/24
Physical activity level	2.80 (0.60)	2.84 (0.52)	3.02 (0.48)
Socioeconomic status	6.83 (1.70)	6.67 (1.42)	7.10 (1.55)
BMI (kg/m^2^)	16.21 (2.22)	16.51 (2.91)	16.21 (2.36)
Aerobic fitness	273.79 (146.74)	313.09 (117.19)	263.63 (128.69)
Gross motor coordination	103.28 (15.41)	106.25 (15.94)	105. 81 (14.64)
**Manipulation check variables**			
Mean step counts during measured time frame*	1506.61 (481.29)	1447.73 (397.73)	838.30 (263.79)
Time spent in MVPA during measured time frame*	15.79%	14.93%	8.43%
RCE*	3.84 (1.65)	2.47 (1.45)	3.25 (1.57)
Pleasure	7.19 (1.18)	7.02 (1.04)	7.47 (1.37)
**Pre and post-test data**			
Pre-test mathematics	48.80 (6.59)	50.83 (6.18)	47.76 (6.10)
Pre-test spelling	54.24 (9.37)	56.46 (6.93)	54.16 (6.98)
Pre-test reading	94.86 (15.21)	102.45 (16.05)	98.33 (14.58)
Post-test mathematics*	53.53 (8.00)	52.76 (6.24)	52.52 (5.48)
Post-test spelling	52.07 (7.20)	52.82 (6.90)	53.39 (7.76)
Post-test reading	101.99 (16.75)	111.67 (18.85)	104.52 (16.48)

BMI = body mass index, RCE = ratings of perceived cognitive engagement, MVPA = moderate-to-vigorous physical activity

*p < .05

**Table 2:** Means and standard deviations in the Flanker task and the Backwards Colour Recall task at pre- and post-test for the three different groups.

	Accuracy *M (SD)*			Reaction Time *M* (*SD*)		
	Combo group	Aerobic group	Cognition group	Combo group	Aerobic group	Cognition group
**Updating**^**a**^						
Pre-test*	11.10 (4.17)	14.18 (3.79)	13.53 (3.94)			
Post-test	14.69 (5.18)	16.42 (3.55)	16.15 (3.09)			
Δ	-3.59 (3.96)	-2.24 (4.48)	-2.62 (4.33)			
**Inhibition**^**b**^						
Pre-test	92.70 (6.64)	94.87 (7.52)	93.18 (5.85)	200.48 (192.17)	128.10 (140.40)	186.43 (154.52)
Post-test	95.38 (5.02)	96.66 (4.05)	96.42 (3.22)	156.92 (246.12)	108.66 (113.21)	111.82 (137.90)
Δ	-2.68 (7.21)	-1.79 (7.64)	-3.24 (5.71)	43.56 (250.42)	19.44 (164.02)	74.60 (135.76)
**Shifting**^**c**^						
Pre-test	87.78 (5.06)	87.77 (7.04)	87.62 (5.97)	477.08 (437.57)	604.63 (421.23)	506.90 (278.57)
Post-test	88.38 (5.15)	89.93 (4.91)	90.00 (3.95)	338.45 (260.48)	499.79 (280.30)	410.12 (203.57)
Δ*^for reaction times^	-0.60 (6.68)	-2.16 (7.67)	-2.39 (6.50)	138.63 (458.62)	104.85 (565.08)	96.78 (283.57)

^a^Updating comprises the number of correct answers in the Backwards Colour Recall task.

^b^Inhibition comprises the percentage of correct answers and the conflict-score between the mean reaction time (in ms) of the incongruent and the congruent trials in the Flanker task.

^c^Shifting comprises the percentage of correct answers and the difference between the mean reaction time (in ms) of the shifting block and the inhibition block.

*p < .05

Δ = the difference of pre- and post-test scores.

**Table 3:** Means and standard deviations in mathematics, spelling and reading at pre- and post-test for the three different groups.

	Accuracy *M (SD)*		
	Combo group	Aerobic group	Cognition group
**Mathematics**			
Pre-test	48.80 (6.59)	50.83 (6.18)	47.76 (6.10)
Post-test	53.53 (8.00)	52.76 (6.24)	52.52 (5.48)
Δ*	-4.73 (4.13)	-1.93 (2.94)	-4.76 (4.02)
**Spelling**			
Pre-test	54.24 (9.37)	56.46 (6.93)	54.16 (6.98)
Post-test	52.07 (7.20)	52.82 (6.90)	53.39 (7.76)
Δ	2.17 (6.73)	3.64 (6.99)	0.77 (6.59)
**Reading**			
Pre-test	94.86 (15.21)	102.45 (16.05)	98.33 (14.58)
Post-test	101.99 (16.75)	111.67 (18.85)	104.52 (16.48)
Δ	-7.13 (9.88)	-9.21 (9.78)	-6.2 (8.22)

*p < .05

Δ = the difference of pre- and post-test scores.

## Results

### Preliminary analyses

At pre-test, ANOVAs revealed no significant group differences in background variables (age, general PA level, socioeconomic status, BMI, aerobic fitness, gross motor coordination,), *F*s(2, 139) ≤ 1.90, n.s., in academic achievement (mathematics, spelling, reading) *F*s(2, 139) ≤ 2.96, n.s., nor in inhibition and shifting *F*s (2, 139) ≤ 2.65, n.s. Only updating revealed a significant group difference at pre-test (*F* = 7.98, *p* = .001, η_p_^2^ = .103). To test whether *physical exertion* in the three experimental conditions differed, the mean step counts were compared between the three groups. The manipulation check analyses revealed significant differences of children’s mean step counts (*F* (2, 139) = 40.75, *p* < .0005, η_p_^2^ = .370) and time spent in moderate-to-vigorous PA (*F* (2, 139) = 31.33, *p* < .0005, η_p_^2^ = .311) during the measured timeframe of 60 minutes. The post-hoc tests revealed that the cognition group had significantly lower physical exertion (mean step counts) compared to both, the combo (*p*s < .0005) and the aerobic group (*p*s < .0005), whereas the combo group and the aerobic group did not differ from each other (*p*s > .386). To check whether the perceived cognitive engagement differed between the three experimental conditions, the perceived cognitive engagement was compared between the three groups. As expected, the three groups differed significantly (*F*(2, 139) = 9.35, *p* < .0005, η_p_^2^ = .119). Post-hoc tests showed that the aerobic group had a significantly lower perceived cognitive engagement compared with the combo group (*p* < .0005) and the cognition group (*p* = .048), whereas the combo group and the cognition group did not differ between each other (*p* = .209). Furthermore, children’s pleasure during the five PA breaks did not differ between the three groups (*F*(2, 139) = 1.67, *p* = .191, η_p_^2^ = .024).

### Main analyses

**Fig 1:**
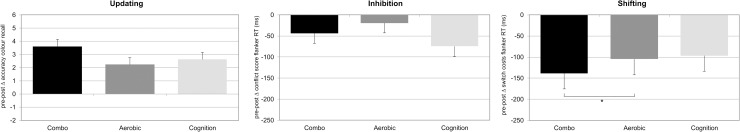
Means and error bars (representing the standard error of the mean) for the change (Δ) in the three core EFs (updating, inhibition, shifting) in the three groups between pre- and post-test. *RT* = reaction time. *p < .05 .

To test the main hypotheses of the study, the three groups were compared regarding the three core EFs between pre- and post-test. The three separate ANCOVAs revealed that updating (*F*(3, 138) = 0.16, *p* = .856, η_p_^2^ = .002) and inhibition (*F*(3, 138) = 0.68, *p* = .507, η_p_^2^ = .010) did not differ significantly between the three groups. However, shifting differed significantly between the groups (*F*(3, 138) = 4.68, *p* = .011, η_p_^2^ = .064). Post-hoc analysis revealed a significantly greater improvement in shifting performance in the combo group than in the aerobic group (*p* = .003), but no significant difference between the combo group and the cognition group (*p* = .176). The aerobic and cognition group did not differ significantly from each other (*p* = .095). The results are depicted in Fig 1.

**Fig 2:**
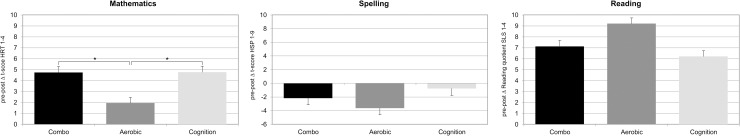
Means and error bars (representing the standard error of the mean) for the change (Δ) in the three academic achievement tests (mathematics, spelling,reading) in the three groups between pre- and post-test. *p < .05.

For academic achievement, only mathematics differed significantly between the three groups (*F*(2, 138) = 7.34, *p* = .001, η_p_^2^ = .096), with post-hoc tests revealing both the combo (*p* = .001) and the cognition group (*p* = .002) to displaying a significantly better mathematic performance when compared against the aerobic group (see Fig 2). The combo group and the cognition group, both characterized as cognitively engaging interventions, did not differ significantly from each other (*p* = .901). No significant differences were found between the three groups in both spelling (*F*(2, 138) = 1.26, *p* = .287, η_p_^2^ = .018) and reading scores (*F*(2, 138) = 1.46, *p* = .236, η_p_^2^ = .021).

## Discussion

The aim of the present study was to investigate the effects of three qualitatively different PA interventions, with distinguishable levels of cognitive engagement and physical exertion, on primary school children’s EFs and academic achievement. It was hypothesized that a PA intervention consisting of both high cognitive engagement and high physical exertion would have a stronger impact on children’s core EFs and academic achievement than a “low” aerobic or a “low” cognitive intervention alone. In summary, the results showed (1) that only the combo intervention (high cognitive engagement and high physical exertion) fostered significant increases in children’s shifting performance, whereas updating and inhibition remained unaffected and (2) that the two cognitively challenging interventions enhanced children’s mathematic performance significantly more than the aerobic intervention. However, spelling and reading performance could not be improved through any of the three interventions. The current results are in line with previous research showing a higher improvement on EFs for those PA interventions with higher amounts of cognitive engagement [23, 68]. In terms of academic achievement, the current results confirm previous studies showing positive effects on children’s mathematic performance after acute PA breaks [69] and long-term classroom-based PA [38].

Considering the existing endurance-orientated literature [70–72] supporting the positive effects of chronic aerobic fitness interventions, an improvement in cognitive function was expected in the aerobic group. Surprisingly, the current results showed that “pure” physical PA breaks with little cognitive effort are not able to enhance specific cognitive components, including the core EFs as well as more global cognitive components, such as academic achievement. This may be explained by the setting of classroom-based PA and its limitations. Due to time and room limitations, compared to physical education and after school programs, classroom-based PA is limited to smaller durations and reduced intensities. Analyses of this current study revealed that only whereas the rest of the time was spent in low to moderate PA. According to Chang et al. [73], heart rates ranging between 70% - 85%, indicating moderate-to-vigorous intensity, seem to benefit cognitive outcomes the most. The suggested range of heart rates of 70% - 85% was hard to reach in this study due to limited space in the classroom. Another plausible explanation is that “pure” aerobic PA does not automatically lead to improved cognitive functions and academic achievement due to the lack of CE in these PA breaks. This explanation is supported by the findings of Schmidt et al. [23], where systematically varying the amount of CE and physical exertion between groups demonstrated no significant improvement in children’s cognitive performance following a “pure” aerobic 6-week intervention, despite their equal increase in aerobic fitness.

According to the cognitive stimulation hypotheses suggested by Tomporowski et al. [18], the improvement in EFs and academic achievement in both of the cognitively engaging intervention groups (combo group and cognition group) was to be expected, along with greater positive effects in the combo group (high CE and physical exertion). The current results confirm the hypotheses showing, that the combo intervention elicited improvements in both children’s shifting and mathematic performance. Surprisingly, the cognition group (high CE, low physical exertion) only demonstrated a beneficial effect in mathematic performance, but no improvement with respect to shifting performance. Results of the manipulation check–showing equally high CE for the combo group as well as for the cognition group–suggest that CE is not the only crucial characteristic responsible for the cognitive gains in terms of EFs. Evidence from an RCT-study [70] including a highly controlled exercise intervention and standardized achievement tests, supports the assumption that different PA intervention types have selective effects on children’s cognitive functions. It seems that EFs are harder to modulate through long-term classroom-based PA interventions compared to academic achievement. This assumption is supported by the meta-analyses of Watson and colleagues [24], showing improvements in academic achievement, yet no effect on cognitive functions, such as EFs, through classroom-based PA. It is likely that only the correct dosage of CE, PA type, duration and intensity enhance children’s cognitive outcomes efficiently.

To bring up a suitable theoretical framework to help explain the current findings, the strength model of self-control, revised by Audiffren and André [74], can be addressed. Self-regulation and EFs share effort as a resource to perform stressful or attentional demanding tasks, such as cognitively challenging PA. If children engage in behaviors requiring a lot of cognitive effort, the cognitive resource is depleted in a subsequent task. However, the training hypotheses postulates an analogy to a trained muscle that the capacity for self-control will decrease after an acute intervention but result in an increased capacity after a long-term intervention. An overload of cognitive capacities performing cognitively engaging PA may lead to an immediate decrease in shifting performance. Referring to the theory of “supercompensation” from the field of applied training science, which describes a decrease in muscle performance immediately after a bout of acute physical exercise but increases in performance after chronic PA, one might speculate that cognitively challenging PA have a similar effect on cognitive functions. Two previous studies [23, 55] investigating acute as well as chronic effects of cognitive engaging PA on children’s EFs may underline these suggestions. Whereas no beneficial effects of acute PA without CE were found, the chronic intervention showed an improved shifting performance after 6 weeks of a physical team game program with a high CE.

The fact that only shifting was positively affected by the combo intervention, while updating and inhibition were not, needs to be discussed in detail regarding the specific content of the intervention. Shifting as a domain of EFs is stimulated when a quick shift between several tasks is required [5]. After counting the total number of required shifting performances inherent in the implemented PA breaks, more shifting performances were required, compared with inhibition and updating actions. It seems that updating and inhibition are harder to implement through a classroom-based PA setting, whereas shifting is likely to be a more immanent dimension of PA.

Both of the cognitively challenging PA interventions (combo group and cognition group) showed an increased mathematic performance after the 20-week intervention. The results, showing only a positive effect for mathematic performance and not for reading and spelling is supported by the review of Donnelly and colleagues [2] concluding that out of four classroom-based PA interventions, three could show a positive effect on children’s mathematic performance. In addition, a recent systematic review and expert panel comes to the conclusion that there is strong evidence for the beneficial effects of PA on mathematics [4]. The obtained result, therefore, is not surprising with respect to the successful enhancement of shifting performance for the combo group. EFs in general are predictors for academic achievement in children and adolescents [75]. Shifting, one of the core EFs, seems to predict academic performance most [8], particularly mathematic performance as shown by Latzman, Elkovitch, Young, and Clark [76] and Yeniad et al. [77]. Shifting is built on updating and inhibition and is strongly related to problem solving, which is a higher-ordered EF [5]. The relationship between shifting and mathematic performance can be explained by improvements in shifting abilities, which are in turn needed to perform different problem-solving strategies, such as flexibly shifting attention to relevant tasks, and moving back and forth between different types of task. Hence, these abilities are required when trying to solve a complex math problem. Although this explanation is quite conceivable and there is some empirical evidence deriving from developmental psychological literature [78, 79] it is hard to adopt it in the present study. The results are showing a better mathematic performance for both cognitively challenging groups (cognition and combo group), but only the combo group improved in terms of shifting performance. One possible explanation could be found in a potential improvement of children’s fine-motor skills (which unfortunately was not measured) through the cognitive intervention. As suggested by Pitchford, Papini, Outhwaite, and Gulliford [80] the influence of fine motor training is correlated with mathematic performance. Finger counting could be the linking mechanism between fine-motor skills and mathematical skills [81, 82].

Like any study, the present study has certain limitations, which need to be addressed. As usual for designs in school-based settings, the randomization of participants was done on a class-level. Further, since time was limited during testing, and the participating children already had large time commitments, the assessment of potential confounders had to be brief. Therefore, we were unable to measure important variables such as disability or language status. However, the teachers were asked in advance whether the participants in their class had any disabilities or language deficits. As a further limitation, it has to be mentioned that each of the three core EF was assessed by only one task. Using multiple tasks per EF component would have been advantageous [83]. However, with respect to ecologically valid implementations and large time commitments for the children involved, it served as a suitable method to test all three core EFs together. However, including all three core EFs is a benefit to give concrete practical advice. In terms of the core EFs, the results support the promotion of shifting performance through classroom-based PA. From a developmental point of view this is a feasible proposal, as shifting is the last EF to be fully developed, and therefore might be more sensitive to positive changes through PA interventions. One might speculate, therefore, that aspects of EFs which are not yet fully developed (as shifting) might be easier to change. This relationship between age and the responsiveness to chronic PA in terms of benefits on EFs needs to be explored in future studies. The implementation of daily PA breaks over a period of 20-weeks is challenging for teachers. The subjective treatment protocols show that the first 10 weeks of intervention, 77% of the breaks were implemented, following by a decrease of 7% in the second half of the intervention. In long-term studies using teachers to implement the interventions over a longer period, treatment fidelity, including direct observation, weekly supervision or periodic meetings [84] are needed and should be considered in the future.

Furthermore, the current study lacks an individually adjusted level of cognitive engagement and physical exertion during the PA breaks. A cognitive under- or overload might be prevented by examining relevant individual characteristics in advance, including sport-specific cognitive expertise, gross motor coordination and aerobic fitness [85]. Consequently, a personally fitted intervention for each child would be possible. However, due to room and time limitations, implementing an individualized cognitive and PA level intervention is a great challenge for future studies, especially for long-term classroom-based PA interventions. Future studies should acknowledge the separate effects of cognitively engaging and cognitively non-engaging interventions, where “cognitively engaging PA” should be defined as a specific type of PA in comparing different PA types in terms of most effective cognitive outputs.

In conclusion, this study adds to the literature on active breaks in schools. A feasible and innovative long-term intervention including cognitive and physical demands was developed and evaluated. Moreover, results revealed that only a combination of long-term PA breaks with high cognitive engagement leads to a stronger improvement in EFs, although not all core EFs seem to be affected equally. Shifting, as one of the core EFs, and its relationship to academic achievement was supported in this present study, where results displayed improvements in mathematic performance. Shifting, therefore, seems to be sensitive to cognitively engaging PA within the preadolescent period, and should be focussed in futures classroom-based PA interventions. Besides physical education, classroom-based PA breaks are a further opportunity, not only to enhance daily PA time, but also to improve children’s cognitive outcomes. High-quality PA, such as a combination of both PA and CE, seems to be the most effective if the adjustment of quantitative characteristics is considered as well.

## Supporting information

S1 Dataset Dataset underlying the findings of the current study. (SAV)
